# Limited knowledge of chronic kidney disease among primary care patients – a cross-sectional survey

**DOI:** 10.1186/1471-2369-13-54

**Published:** 2012-07-02

**Authors:** Wai Leng Chow, Veena D Joshi, Aung Soe Tin, Saskia van der Erf, Jeremy Fung Yen Lim, Teck Sin Swah, Stephanie Swee Hong Teo, Paul Soo Chye Goh, Gilbert Choon Seng Tan, Crystal Lim, Terence Yi-shern Kee

**Affiliations:** 1 Centre for Health Services Research, Singapore Health Services, 168 Jalan Bukit Merah, #06-08 Tower 3, Singapore, 150169, Singapore; 2 Department of Strategic Research, National Health Insurance Fund France, Paris, France; 3 Lien Centre for Palliative Care, Duke-NUS, Singapore, Singapore; 4 SingHealth Polyclinics, Bedok, Singapore, Singapore; 5 SingHealth Polyclinics, Tampines, Singapore, Singapore; 6 SingHealth Polyclinics, Geylang, Singapore, Singapore; 7 Singapore General Hospital, Singapore, Singapore; 8 Department of Renal Medicine, Singapore General Hospital, Singapore, Singapore

**Keywords:** Chronic kidney disease, General knowledge, Influencing factors, Primary care

## Abstract

**Background:**

Kidney disease is the 9th leading cause of death in Singapore. While preventive effects have focused on early detection and education, little is known about the knowledge level of chronic kidney disease (CKD) locally. We seek to evaluate the knowledge of CKD among primary care patients.

**Methods:**

We conducted a cross-sectional survey of a convenience sample of 1520 patients from 3 primary care centers. Those with existing CKD or on dialysis were excluded. Knowledge was assessed based on 7 questions on CKD in the self-administered questionnaire. One point was given for each correct answer with a maximum of 7 points.

**Results:**

1435 completed all 7 questions on CKD. Mean age was 48.9 ±15.0 (SD) years. 50.9% were male. 62.3% had a secondary and below education and 52.4% had a monthly household income of ≤ $2000. 43.7% had chronic diseases. Mean score was 3.44 ± 1.53 (out of a maximum of 7). Median score was 4. In multivariate logistic regression, being older {>60 years [Odds Ratio (OR) 0.50, 95% Confidence Interval (CI) 0.32-0.79]; 40–60 years (OR 0.62, 95% CI 0.43,0.89)}, less educated [up to primary education (OR 0.33, 95% CI 0.22-0.49)], having a lower monthly household income [<S$2000 (OR 0.41, 95% CI 0.26-0.66); S$2000-4999 (OR 0.53, 95%CI, 0.33-0.83)], and being non-professionals [OR 0.66, 95% CI 0.43-0.99] (all p < 0.05)] were likely to score less.

**Conclusion:**

This suggests that CKD education should be targeted at older patients with lower education and lower socioeconomic status.

## Background

Chronic kidney disease (CKD) is the 9^th^ leading cause of death in Singapore in 2008 [1]. In the sixth report by the Singapore Renal Registry 2008/2009, diabetes and hypertension accounted for 63.9% and 10.7% of new kidney failure respectively. The incidence of chronic kidney disease has been on the rise both locally in Singapore as well as globally. In 2008, there were 332.7 new cases of end stage renal disease (ESRD) per million resident population (crude rate) up from 213.2 per million resident population in 1999 [2]. This increasing trend is also seen in the United States. In 2009 alone, the incidence of ESRD was reported as 355 per million population [3].

CKD prevention internationally has been focused on screening programs that increase awareness and early detection of CKD among at risk populations. The Kidney Early Evaluation Program (KEEP) in the United States encourages those with diabetes, hypertension or an immediate relative with a history of diabetes, hypertension or kidney disease to go for screening. Education on CKD is provided as part of the program [4].

In Singapore, the National Kidney Foundation of Singapore addresses CKD prevention through primary, secondary and tertiary preventive strategies. They include public education, screening for kidney disease and associated chronic diseases, disease management program as well as the optimization of care of patients at risk for kidney disease through Prevention Centers. The Prevention Program was initiated in 1997 [5].

Education to improve knowledge plays an important role in CKD prevention regardless of whether it is primary, secondary or tertiary prevention. Knowledge of how an action impacts an individual’s health, particularly if it involves modification of lifestyle habits, is a prerequisite for behavior change to occur [6].

However, while much has been documented on the successes of screening programs in increasing awareness of CKD both locally and internationally, there is limited research into the actual knowledge level of CKD among the general population.

Our study seeks to evaluate the knowledge level of CKD of the primary care patients in Singapore.

## Methods

### Sample population

This was a cross-sectional survey of a convenience sample of 1520 consecutive members of the public seeking attendance at 3 primary care public medical centers in Singapore. Those who were below the age of 21 years, with pre-existing CKD, on dialysis or a history of kidney transplant were excluded. (See Annex 1 for screening questions). Trained surveyors approached members of the public in the primary care centres at the public waiting areas. The survey was mainly self-administered. Trained surveyors were at hand to provide any clarification and assistance when required. Respondents were given the option of answering the survey in English, Chinese or Malay. Ethics approval was given by SingHealth Centralized Institutional Review Board.

### Questionnaire development

The questionnaire consisted of three main domains: 1) attitudes toward living organ donation; 2) knowledge of CKD; and 3) demographics. Pre-testing of the questionnaire developed by the team was performed with members of the public through focus group discussions. The questionnaire was tested for face validity as well as content saturation. Questions pertaining to knowledge of CKD were also specifically tested for readability and content validity to help determine the level of difficulty of the written material. The questionnaire was refined using feedback from the focus group discussions and expert review by nephrologists and primary care physicians. The questionnaires were subsequently translated into and back translated from Chinese and Malay.

The self-administered questionnaire included 7 questions to assess the knowledge of CKD and additional questions on demographics. The questions were developed using a combination of patient references for CKD [7,8] as well as in consultation with nephrologists and primary care physicians. The questions were close-ended single response type multiple choice questions with the intention of assessing the knowledge of the respondents in the following 7 domains: 1) Anatomy, 2) Physiology (function of the kidney), 3) Etiology of CKD; 4) Symptoms of early CKD, 5) Progression, 6) Treatment of end stage renal failure and 7) Resource available to CKD patients. Respondents were asked the following 7 questions and were asked to choose the best option to each question (See Annex 2):

1. How many healthy kidney(s) does a person need to lead a normal life? (Anatomy)

2. What is the function of a kidney in a human body? (Physiology)

3. What can cause kidney disease? (Etiology)

4. What are the symptoms of early kidney disease that might progress to kidney failure? (Presentation)

5. Which of the following statement about kidney disease is INCORRECT: (Progression)

6. Where can dialysis treatment be carried out? (Resources available)

7. What is the best medical treatment for End Stage Kidney Failure? (Treatment)

Information obtained on the demographic profile of respondents included data on age, education level, average monthly household income as well as self-reported presence of comorbidities.

### Data analysis

Respondents were categorized into younger than 40 years old, 40–60 years old and above 60 years old. They were also grouped into professionals and non-professionals. Included under non-professionals were respondents who were working on a flexi-hour basis, unemployed and retirees.

One point was given for each correct answer to each question on CKD, giving a maximum possible score of 7 points and a minimum of 0 points. Respondents were considered to have average overall knowledge of CKD if they scored at least the median score i.e. ≥ 4 points.

Independent sample t-test, chi-square test and multiple logistic regression analyses were performed to examine the differences and associations.

By treating ≥4 and <4 scores as dependent dichotomous variable, we used chi-square tests to evaluate the associations with each demographic variable during bivariate analysis. We then carried out multivariate logistic regression analysis to adjust for potential confounders for demographic variables that were significantly (p-value <0.05) associated in bivariate analyses.

Individual questions/domains were also treated as dependent dichotomous variable (“Correct” or “Incorrect” answer) and associations with each demographic variable were evaluated with chi-square tests during bivariate analysis. Seven multiple logistic regression models were used to adjust for potential confounding variables and examine the independent factors associated with the respective domains of knowledge. Factors that were found to be significantly associated (p-value <0.05) with knowledge in bivariate analysis were included in the models.

These analyses were performed to examine if there were any differences in the factors associated with overall knowledge versus knowledge in the different domains of CKD.

Data cleaning, coding and analyzing were performed using the Statistical Package for Social Sciences (SPSS) 15 for Windows version. The level of statistical significance was set at p < 0.05.

## Results

Our sample respondents had similar percentage distribution to the national population in terms of gender and race. Respondents with secondary education were over represented in our sample as compared to the national population (40.7% versus 32.2%). (Table 1)

**Table 1:** Demographic characteristics of sample in comparison with national population (N = 1435)

	Sample characteristics No (%)	National statistics (End June 2009) (%)
**Gender**		
Male	727 (50.9%)	49.4%
Female	701 (49.1%)	50.5%
**Race**		
Chinese	1022 (71.3%)	74.2%
Malay	225 (15.7%)	13.4%
Indian	145 (10.1%)	9.2%
Others	42 (2.9%)	3.2%
**Education**		
Up to primary	21.6%	27.6%
Secondary	40.7%	32.2%
A-level and above	37.6%	40.2%

Reference: Yearbook of Statistics Singapore 2010, Department of Statistics, Singapore [9].

Of the 1520 respondents, 1435 (94.4%) respondents answered all the 7 questions on knowledge of CKD. This data were further analyzed.

### Demographic characteristics of the respondents

The average age of the respondents was 48.9 ±15.0 years. 50.9% were men. 40.7% and 37.6% had secondary level and above secondary level of education respectively. 52.6% of respondents had a household monthly income of < S$2000. Only 15.8% of the respondents worked as professionals or executives. 25.5% were working in production or clerical jobs or were self-employed. 30.9% were not employed, retired or housewives and 18.0% were working as part-timers or on a flexi-hour basis (others category). Christians (19.7%) and Muslims (21.3%) each made up about 20% of the respondents. 31.2% were Buddhists while 27.5% were believers of other religions. 43.7% of the respondents had co-existing chronic diseases such as diabetes, hypertension, high cholesterol, ischemic heart disease and stroke.

### Respondents’ knowledge on chronic kidney disease

**Figure 1:**
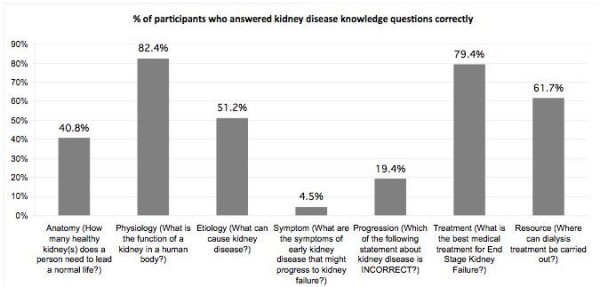
Percentage distribution of correct answers in different knowledge domains of chronic kidney disease.

Majority (94.4%) of the respondents answered all the questions on general knowledge on chronic kidney disease. Of these respondents, 82.4% knew the function of the kidney is to filter waste products in the blood and 79.4% knew that kidney transplant is the best treatment for end stage renal failure. 61.7% knew that dialysis treatment can be carried out either at home or at a dialysis centre. However, only 51.2% knew that kidney disease can be caused by hypertension, diabetes and inherited conditions. 40.8% of the respondents knew that only one kidney is needed for a human being to live a normal life. Only 4.5% knew that early kidney disease could present without any symptoms or complaints and 19.4% correctly identified that CKD cannot be cured with medications. (Figure 1)

**Figure 2:**
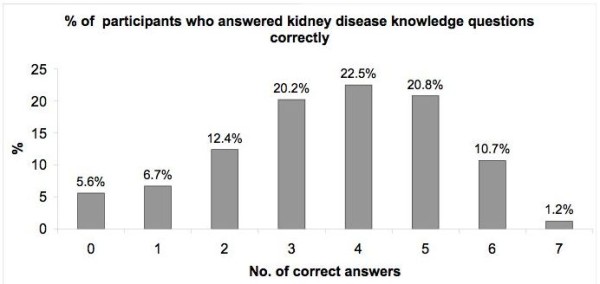
Percentage distribution of correct answers on knowledge questions on chronic kidney disease.

Majority of the respondents answered 3 to 5 questions correctly giving a mean score of 3.44 ± 1.53 and a median score of 4. (Figure 2) Eighty (5.6%) respondents had no knowledge of kidney disease. Of these 80 respondents, most of them were > 40 years old (72.1%), were Buddhists (31.5%), had monthly household income of < S$2000 (52.3%), had below secondary education (78.1%), were non professionals (84.2%) and had co-existing chronic disease (44.3%).

### Defining overall knowledge and associated factors

Four points reflected the average knowledge level. Therefore in this study, a respondent was deemed to have an average knowledge of CKD if at least a median score was achieved i.e. ≥ 4 points out of a maximum possible score of 7 points. (Figure 2)

There were significantly more respondents who were above 60 years of age, non-Chinese, had below secondary level education, a monthly household income of below S$2000, were non-professionals and believers of Islam, as compared to other religions, who were less knowledgeable in CKD (all p < 0.001). It was observed that respondents who did not have chronic disease and who had no children were more knowledgeable as compared to respondents who had chronic disease (p =0.038) and who had children (p < 0.001). There was no significant difference in knowledge level observed by gender. (Table 2)

**Table 2:** Demographic characteristics of patients by number of correct answers on kidney disease related knowledge questions

	**<4 correct answers**	**≥4 correct answers**	**Total (N = 1435)**	p-value*
	**No (%)**	**No (%)**	**No**	
**Age (year)**				<0.001
< 40	144 (35.9)	257 (64.1)	401	
40-60	319 (45.9)	376 (54.1)	695	
> 60	193 (56.9)	146 (43.1)	339	
**Gender**				0.550
Male	331 (45.5)	396 (54.5)	727	
Female	320 (45.6)	381 (54.4)	701	
Missing			7	
**Ethnicity**				<0.001
Chinese	425 (41.6)	597 (58.4)	1022	
Malay	122 (54.2)	103 (45.8)	225	
Indian	90 (62.1)	55 (37.9)	145	
Others	19 (45.2)	23 (54.8)	42	
Missing			1	
**Education**				<0.001
Up to primary	205 (66.3)	104 (33.7)	309	
Secondary	293 (50.3)	289 (49.7)	582	
Above secondary	154 (28.6)	384 (71.4)	538	
Missing			6	
**Occupation**				<0.001
Professional and executive	50 (22.0)	177 (78.0)	227	
Non-Professional	601 (50.1)	598 (49.9)	1199	
Missing			9	
**Income (S$)**				<0.001
< 2000	391 (55.7)	311 (44.3)	702	
2000 - 4999	169 (37.2)	285 (62.8)	454	
≥ 5000	35 (19.1)	148 (80.9)	183	
Missing			96	
**Marital status**				<0.001
Married	499 (47.1)	561 (52.9)	1060	
Single	108 (37.0)	184 (63.0)	292	
Divorced /separated/Widowed	49 (59.0)	34 (41.0)	83	
Missing			100	
**Religion**				
Buddhism #	213 (47.0)	240 (53.0)	453	<0.001
Christianity **	95 (33.3)	190 (66.7)	285	
Islam	160 (54.6)	133 (45.4)	293	
Others	187 (46.8)	213 (53.3)	400	
Missing			4	
**Nationality**				<0.001
Singapore citizen	593 (44.3)	745 (55.7)	1338	
Permanent resident	55 (65.5)	29 (34.5)	84	
Foreigner	6 (75.0)	2 (25.0)	8	
Missing			15	
**Presence of children**				0.038
Yes	302 (48.9)	316 (51.1)	618	
No	348 (43.3)	455 (56.7)	803	
Missing			14	
**Presence of chronic diseases**				0.007
Yes	499 (47.9)	543 (52.1)	1042	
No	124 (39.2)	192 (60.8)	316	
Missing			77	

*All tests are carried out using chi-square tests.

# Buddhists were significantly (p =0.003) more knowledgeable than Muslims.

** Christians were significantly more knowledgeable than Muslims (p < 0.0001) and Others (p = 0.002).

A total of 82 (5.7%) respondents scored ≥ 6 points out of 7 points i.e. ≥ 85% and their demographic characteristics were exactly the same as those who scored ≥ 4.

### Knowledge of different domains of chronic kidney disease and associated factors

Of the 7 domains covered in the questionnaire, Physiology had the highest percentage of correct responses, followed by Treatment, Resource available, Etiology, Anatomy, Progression and Symptom.

Having a primary and below education, being a non-professional, and having an income of below S$ 2000 were associated with higher percentage of incorrect responses across all 7 domains. Being above 60 years old was associated with incorrect response across all domains except in Symptom. (See Tables 3 and 4 for details.)

**Table 3:** Relationships between different domains of knowledge and demographics as well as clinical characteristics in both bivariate analysis and multiple logistic regression

	**Anatomy (n = 1498)**			**Physiology (n = 1497)**			**Etiology (n = 1499)**		
	[How many healthy kidney(s) does a personneed to lead a normal life?]			[What is the function of a kidney in a human body?]			[What can cause kidney disease?]		
	Correct	Incorrect	OR (95% CI)	Correct	Incorrect	OR (95% CI)	Correct	Incorrect	OR (95% CI)
Frequency (%)	611 (40.8)	887		1234 (82.4)	263		767 (51.2)	732	
Age: mean(SD), year	46.6 (14.9) **	50.6 (15.1)		48.4 (14.9)^	51.2 (15.9)		46.1 (14.6)**	51.9 (15.2)	
Gender, freq (%)									
Male	324 (43.1)	428 (56.9)		620 (82.6)	131 (17.4)		378 (50.3)	373 (49.7)	
Female	285 (38.7)	452 (61.3)		608 (82.5)	129 (17.5)		386 (52.3)	352 (47.7)	
Ethnicity, freq (%)									
Chinese	465(44.0)	591 (56.0)		907 (86.0)	148 (14.0)		557 (52.6)	502 (47.4)	
Malay	70 (28.5)	176 (71.5)		177 (71.4)**	71 (28.6)		125 (51.4)	118 (48.6)	
Indian	55 (37.2)**	93 (62.8)		114 (77.0)	34 (23.0)		61 (40.9)	88 (59.1)	
Others	21 (46.7)	24 (53.3)		34 (79.1)	9 (20.9)		24 (54.5)	20 (45.5)	
Citizenship, freq (%)									
Singaporean	577 (41.4)	818 (58.6)		1152 (82.6)	242 (17.4)		724 (52.0)	669 (48.0)	
Others (Permanent Residents and Foreigners)	32 (33.3)	64 (66.7)		77 (80.2)	19 (19.8)		41 (41.8)	57 (58.2)	
Education, freq (%)									
Up to primary	84 (26.0)**	239 (74.0)	0.4 (0.3-0.6)	214 (66.3)**	109 (33.7)	0.2 (0.1-0.4)	114 (35.3)**	209 (64.7)	0.4 (0.3-0.6)
Secondary	213 (34.6)	403 (65.4)	0.6 (0.4-0.8)	509 (82.8)	106 (17.2)	0.5 (0.3-0.8)	298 (48.3)	319 (51.7)	0.7 (0.5-0.9)
Above Secondary	313 (56.8)	238 (43.2)	Reference	506 (91.8)	45 (8.2)	Reference	352 (64.0)	198 (36.0)	Reference
Occupation, freq (%)									
Professional & Exec	148 (63.2)	86 (36.8)	Reference	208 (89.3)	25 (10.7)		159 (67.7)	76 (32.3)	
Non-professional	460 (36.8)**	790 (63.2)	0.6 (0.5-0.9)	1015 (81.2)*	235 (18.8)		602 (48.2)**	647 (51.8)	
Income (SGD), freq (%)									
<$2000	233 (32.0)**	495(68.0)	0.6 (0.4-0.9)	727 (77.6)**	163 (22.4)		340 (46.6)**	389 (58.4)	
$2000-$4999	232 (48.7)	244 (51.3)	0.8 (0.6-1.2)	417 (87.6)	59 (12.4)		261 (54.9)	214 (45.1)	
> = $5000	113 (61.1)	72 (38.9)	Reference	171 (92.9)	13 (7.1)		122 (65.9)	63 (34.1)	
Having Children, freq (%)									
Yes	425 (39.0)*	661 (61.0)		896 (82.4)	192 (17.6)		534 (49.0)^	556 (51.0)	
No	154 (47.2)	172 (52.8)		266 (81.6)	60 (18.4)		184 (56.6)	141 (43.4)	
Having comorbidity, freq (%)									
Yes	245 (40.3)	363 (59.7)		499 (81.9)	110 (18.1)		282 (46.5)*	325 (53.5)	
No	332 (42.0)	459 (58.0)		651 (82.4)	139 (17.6)		430 (54.2)	363 (45.8)	
Religion, freq (%)									
Buddhism	182 (38.8)	287 (61.2)		387 (82.7)	81 (17.3)		243 (51.7)	227 (48.3)	
Christianity	148(49.8)	149 (50.2)		272 (91.9)	24 (8.1)		162 (54.4)	136 (45.6)	
Islam	94 (30.1)**	218 (69.9)		226 (72.0)**	88 (28.0)		156 (50.3)	154 (49.7)	
Others	185 (44.8)	228 (55.2)		343 (83.3)	69 (16.7)		203 (49.0)	211 (51.0)	
Age group, freq (%)									
< 40 years	207 (50.6)	202 (49.4)		344 (84.1)	65 (15.9)		257 (63.0)	151 (37.0)	Reference
40 – 60 years	284 (39.0)	444 (61.0)		612 (84.1)	116 (15.9)		369 (50.5)	362 (49.5)	0.7 (0.6-1.0)
> 60 years	119 (33.2)**	239 (66.8)		277 (77.6)^	80 (22.4)		140 (39.3)**	216 (60.7)	0.6 (0.4-0.9)
Marital status, freq (%)									
Married	433 (39.1)	675 (60.9)		923 (83.3)	185 (16.7)		545 (49.2)^	562 (50.8)	
Single	142 (46.9)	161 (53.1)		252 (83.2)	51 (16.8)		178 (58.6)	126 (41.4)	
Divorced/ Separated /Widowed	35 (41.7)	49 (58.3)		57 (68.7)**	26 (31.3)		43 (51.2)	41 (48.8)	

*OR* Odds ratio, *CI* confidence interval, *SD* standard deviation.

OR derived from multiple logistic regression (MLR) after adjusting for potential confounders. MLR is performed using dependent variables as knowledge and independent variables that are significantly associated with knowledge in bivariate analysis. [**p < 0.001, *p < 0.01, ^p < 0.05: These p-values are derived from bivariate analysis].

**Table 4:** Relationships between different domains of knowledge and demographics as well as clinical characteristics in both bivariate analysis and multiple logistic regression.

	**Symptom (n = 1495)**			**Progression (n = 1462)**			**Treatment (n = 1501)**			**Resource Available (n = 1503)**		
	[What are the symptoms of early kidney disease that might progress to kidney failure?]			[Which of the following statement about kidney disease is INCORRECT:]			[What is the best medical treatment for End Stage Kidney Failure?]			[Where can dialysis treatment be carried out?]		
	Correct	Incorrect	OR (95% CI)	Correct	Incorrect	OR (95% CI)	Correct	Incorrect	OR (95% CI)	Correct	Incorrect	OR (95% CI)
Frequency (%)	67 (4.5)	1428		284	1178		1192	309		928	575	
Age: mean(SD), year	44.1 (14.9)*	49.1 (15.1)		50.0**	44.4		48.0**	52.5		47.9*	50.5	
Gender, freq (%)												
Male	40 (5.3)	711 (94.7)		141 (19.1)	598 (80.9)		583 (77.4)	170 (22.6)		453 (60.0)	302 (40.0)	
Female	26 (3.5)	708 (96.5)		141 (19.7)	573 (80.3)		604 (81.6)	136 (18.4)		471 (63.8)	267 (36.2)	
Ethnicity, freq (%)												
Chinese	48 (4.5)	1009 (95.5)		221 (21.4)	812 (78.6)		873 (82.5)	185 (17.5)		709 (67.0)	349 (33.0)	
Malay	10 (4.1)	233 (95.9)		42 (17.9)	193 (82.1)		179 (72.2)	69 (27.8)		122 (49.4)	125 (50.6)	
Indian	5 (3.4)	143 (96.6)		14 (9.5)*	133 (90.5)		102 (68.9)**	46 (31.1)		67 (45.0)**	82 (55.0)	
Others	3 (7.0)	40 (93.0)		6 (13.6)	38 (86.4)		36 (80.0)	9 (20.0)		27 (60.0)	18 (40.0)	
Citizenship, freq (%)												
Singaporean	64 (4.6)	1327 (95.4)		270 (19.9)	1088 (80.1)		1113 (79.6)	285 (20.4)		886 (63.4)	511 (36.6)	Reference
Others (Permanent Residents and Foreigners)	2 (2.1)	94 (97.9)		12 (12.4)	85 (87.6)		74 (76.3)	23 (23.7)		39 (39.8)**	59 (60.2)	0.3 (0.2-0.6)
Education, freq (%)												
Up to primary	8 (2.5)^	313 (97.5)		33 (10.6)**	279 (89.4)	0.5 (0.3-0.9)	227 (70.3)**	96 (29.7)	0.6 (0.4-0.9)	157 (48.6)**	166 (51.4)	0.5 (0.3-0.7)
Secondary	24 (3.9)	591 (96.1)		104 (17.4)	494 (82.6)	0.9 (0.6-1.3)	488 (78.7)	132 (21.3)	0.8 (0.5-1.2)	377 (61.0)	241 (39.0)	0.7 (0.5-0.9)
Above Secondary	34 (6.2)	516 (93.8)		146 (26.8)	398 (73.2)	Reference	472 (85.7)	79 (14.3)	Reference	390 (70.5)	163 (29.5)	Reference
Occupation, freq (%)												
Professional & Exec	18 (7.7)	217 (92.3)		65 (27.9)	168 (72.1)		200 (86.2)	32 (13.8)		170 (72.3)	65 (27.7)	
Non-professional	48 (3.9)^	1197 (96.1)		218 (18.0)**	996 (82.0)		980 (78.2)*	273 (21.8)		750 (59.9)**	503 (40.1)	
Income (SGD), freq (%)												
<$2000	29 (4.0)^	697 (96.0)		107 (15.0)**	608 (85.0)	0.5 (0.3-0.8)	544 (74.5)**	186 (25.5)		412 (56.4)**	319 (43.6)	
$2000-$4999	19 (4.0)^	458 (96.0)		105 (22.8)	356 (77.2)	0.7 (0.5-1.0)	414 (86.8)	63 (13.2)		326 (68.3)	151 (31.7)	
> = $5000	15 (8.2)	169 (91.8)		56 (30.9)	125 (69.1)	Reference	161 (87.0)	24 (13.0)		139 (75.1)	46 (24.9)	
Having Children, freq (%)												
Yes	39 (3.6)*	1047 (96.4)		179 (16.9)**	880 (83.1)		866 (79.4)	225 (20.6)		655 (60.1)	434 (39.9)	
No	23 (7.1)	301 (92.9)		94 (29.4)	226 (70.6)		263 (80.4)	64 (19.6)		209 (63.7)	119 (39.0)	
Having comorbidity, freq (%)												
Yes	22 (3.6)	582 (96.4)		91 (15.4)*	500 (84.6)		457 (78.0)	134 (22.0)		372 (61.3)	235 (38.7)	
No	40 (5.1)	751 (94.9)		177 (22.9)	597 (77.1)		641 (80.9)	151 (19.1)		484 (60.9)	311 (39.1)	
Religion, freq (%)												
Buddhism	12 (2.6)^	458 (97.4)		92 (20.2)	364 (79.8)		373 (79.5)	96 (20.5)		300 (64.0)	169 (36.0)	
Christianity	12 (4.1)	283 (95.9)		74 (25.3)	218 (74.7)		258 (86.6)	40 (13.4)		212 (71.1)	86 (28.9)	
Islam	13 (4.2)	297 (95.8)		50 (16.6)	252 (83.4)		225 (71.7)**	89 (28.3)		161 (51.3)**	153 (48.7)	
Others	29 (7.0)	384 (93.0)		67 (16.5)^	339 (83.5)		330 (79.9)	83 (20.1)		251 (60.5)	164 (39.5)	
Age group, freq (%)												
< 40 years	25 (6.2)	381 (93.8)		108 (26.8)	295 (73.2)		342 (83.4)	68 (16.6)	Reference	265 (64.6)	145 (35.4)	
40 – 60 years	30 (4.1)	701 (95.9)		132 (18.5)	580 (81.5)		598 (81.9)	132 (18.1)	0.9 (0.6-1.3)	464 (63.3)	269 (36.7)	
> 60 years	11 (3.1)	343 (96.9)		43 (12.5)**	301 (87.5)		251 (70.1)**	107 (29.9)	0.5 (0.3-0.8)	196 (55.1)^	160 (44.9)	
Marital status, freq (%)												
Married	41 (3.7)	1063 (96.3)		190 (17.7)	886 (82.3)		883 (79.5)	227 (20.5)		672 (60.6)	436 (39.4)	
Single	20 (6.6)	283 (93.4)		81 (26.9)	220 (73.1)		246 (81.2)	57 (18.8)		209 (68.1)	98 (31.9)	
Divorced/ Separated /Widowed	5 (6.0)	79 (94.0)		13 (15.7)*	70 (84.3)		61 (71.8)	24 (28.2)		45 (53.6)^	39 (46.4)	

*OR* Odds ratio, *CI* confidence interval, *SD* standard deviation.

OR derived from multiple logistic regression (MLR) after adjusting for potential confounders. MLR is performed using dependent variables as knowledge and independent variables that are significantly associated with knowledge in bivariate analysis. **p < 0.001, *p < 0.01, ^p < 0.05 [These p-values are derived from bivariate analysis.].

### Association between knowledge and demographic characteristics – Regression analysis

Multivariate logistic regression, where dichotomous dependent variable was overall knowledge level (i.e., knowledge score <4 and score > =4), revealed being older {above 60 years [Odds Ratio (OR) 0.50, 95% Confidence Interval (CI) 0.32,0.79] and 40–60 years (OR 0.62, 95% CI 0.43,0.89)}, respondents with lower education [up to primary education (OR 0.33, 95% CI 0.22-0.49)] and respondents with lower monthly family income [<S$2000 (OR 0.41, 95% CI 0.26,0.66 and S$2000-4999 (OR 0.53, 95% CI 0.33,0.83)], respondents who worked as non-professional (OR 0.66, 95% CI 0.43,0.99) and permanent residents (OR 0.27, 95% CI 0.15,0.47) (all p < 0.05) were more likely to have a lower knowledge level. Only significant variables are presented in Table 5.

**Table 5:** Multivariate logistic regression analysis to determine socio-demographic factors that influences the knowledge levels

	B	S.E.	Sig.	Odds Ratio (OR)	95.0% C.I.for OR	
					Lower	Upper
Singapore Permanent Resident	−1.327	0.297	0.000	0.265	0.148	0.474
Foreigners	−1.018	1.113	0.361	0.361	0.041	3.202
Singapore Citizen (Ref)						
40 - 60 yrs	−0.480	0.186	0.010	0.619	0.430	0.890
> 60 yrs	−0.695	0.234	0.003	0.499	0.316	0.789
< 40 yrs (Ref)						
Up to primary	−1.125	0.209	0.000	0.325	0.216	0.489
Secondary	−0.651	0.167	0.000	0.521	0.376	0.723
Above secondary (Ref)						
< S$2000	−0.881	0.240	0.000	0.414	0.259	0.663
S$2000-4999	−0.641	0.233	0.006	0.527	0.334	0.831
> = S$5000 (Ref)						
Non professional	−0.421	0.210	0.045	0.656	0.435	0.990
Professional & Exec (Ref)						

Dependent variable is dichotomous outcome of <4 and ≥4 knowledge scores. Only significant variables are displayed.

The seven multiple logistic regression models for the respective 7 knowledge domains revealed that after adjusting for potential confounding demographic factors, having a below secondary level education as compared to above secondary education, was the only factor that was significantly associated with knowledge across all domains except Symptom [Anatomy, up to primary education OR 0.4 (0.3-0.6) and secondary education OR 0.6 (0.4-0.8); Physiology, up to primary education OR 0.2 (0.1-0.4) and secondary education OR 0.5 (0.3-0.8); Etiology, up to primary education OR 0.4 (0.3-0.6) and secondary education OR 0.7 (0.5-0.9); Progression, up to primary education OR 0.5 (0.3-0.9); Treatment, up to primary education OR 0.6 (0.4-0.9); Resource available, up to primary education OR 0.5 (0.3-0.7) and secondary education OR 0.7 (0.5-0.9)].

Lower income (<S$ 2000) was significantly associated with lower knowledge in Anatomy [OR 0.6 (0.4-0.9)] and Progression [OR 0.5 (0.3-0.8)] as compared with those with an income of S$5000 or more. Being older (>60 years old) was significantly associated with lower knowledge in Etiology [OR 0.6 (0.4-0.9)] and Treatment [OR 0.5 (0.3-0.8)] as compared to those below 40 years old. Being a non-professional was only associated with poorer knowledge in Anatomy [OR 0.6 (0.5-0.9)] and being a non-Singaporean was associated with reduced knowledge in Resource availability [OR 0.3 (0.2-0.6)]. (See Tables 3 and 4 for details.)

The 2 regression analyses revealed that overall knowledge levels as well as knowledge in respective domains were associated with education level, income as well as age group.

## Discussion

The incidence of chronic kidney disease (CKD) is on the rise globally. Existing prevention programs have focused on increasing awareness of CKD both among the general population as well as the at-risk population through public education. However, little is known of the actual CKD knowledge levels of these populations. Our study suggests that only about 51% of primary care patients who are not known to have CKD knew that CKD could be caused by diabetes, hypertension and hereditary conditions. Less than 50% of respondents knew only one kidney was required to maintain a normal life; early CKD can present asymptomatically and CKD was not curable with medications. Additionally, only about 5% of respondents knew that early kidney disease could present without any symptoms or complaints. Older respondents who had below primary level education, a monthly household income of < S$2000 and were non-professionals were more likely to have lower knowledge levels on chronic kidney disease. This suggests that a more targeted approach could be taken to address the reduced knowledge level of CKD among primary care patients.

In Singapore, the National Kidney Foundation (NKF) Singapore Prevention Programme in addition to increasing awareness of CKD, focused on primary prevention by public education and screening for chronic conditions such as diabetes and hypertension. This is aimed to intervene prior to the onset of any evidence of kidney disease [10]. However, despite the existence of the program since 1997, the incidence of CKD is still increasing locally [2]. This could be attributed in part to the increasing incidence and prevalence of population with diabetes, the leading cause of CKD in Singapore, and hypertension as well as an aging population [11].

We found that about 55% of respondents had average knowledge (defined as a score of ≥4 out of possible 7 points) in general knowledge questions related to kidney function, symptoms, risk factors and treatment options. However, less than half of the respondents correctly identified that only one kidney was required for a normal life and could not be cured with medications. More importantly, less than 10% of respondents correctly answered that early CKD presented asymptomatically. In a study by Lam et al that compared knowledge levels of kidney disease between the general public and health care providers, the general public was similarly found to be less knowledgeable in some aspects of kidney disease. Fewer general public respondents knew only one kidney was required for a normal life and that CKD could progress without symptoms for many years [12].

Only 51.3% of our respondents could correctly identify all the risk factors for developing CKD, namely diabetes, hypertension and hereditary conditions. Similar findings were reported in a survey carried out in Australia among respondents with diabetes and associated risk factors. Few identified diabetes (8.6%) and hypertension (2.8%) as risk factors for developing kidney disease when asked to respond to an open-ended question on risk factors. At the same time, only 21% identified 3 or more factors [13].

While demographic factors have been suggested by other studies to influence knowledge of other chronic diseases, none have explored the relationship between demographic factors and their influence on knowledge of chronic kidney disease among the general primary care patient population. We found that younger age, having an above primary education level, being a professional and having a monthly household income of ≥ S$2000 were associated with better knowledge of CKD. A monthly household income of < S$2000 represented approximately, the lowest 20^th^ centile income among the Singapore population [14]. In a cross-sectional survey of the American general population by Ayotte et al on hypertension knowledge among the respondents, ethnicity, gender and education level were found to be associated with knowledge levels [15]. Knowledge of CKD was not associated with ethnicity and gender in our study. Fezeu et al have reported age and education to be associated with knowledge on diabetes. At the same time, having a relative with chronic disease was associated with better knowledge [16].

We found patients with chronic diseases who are at higher risk of developing CKD to have lower overall knowledge levels than those without chronic disease in our bivariate analysis. Although this association was no longer significant in the multivariate analysis, this suggests that there might be a need for more targeted and tailored education to increase awareness particularly among this high- risk group in order for successful secondary prevention or early detection of CKD. Petrella et al have reported hypertension patients to be unaware of the association of hypertension and chronic kidney disease. They also had limited knowledge of lifestyle issues affecting hypertension control [17]. In another survey of high-risk patients with hypertension and diabetes seen in primary care practices, Boulware et al found that only 20% of the respondents felt that they were “very likely” to develop CKD and about 33% were “very concerned” about developing CKD in 10 years. At the same time, females and those with lower health literacy had lower perceived susceptibility of developing CKD [18].

Knowledge of chronic kidney disease underpins the success of disease prevention as described by the health belief model [6]. Without adequate basic knowledge of risk factors for developing CKD, and that early CKD can present asymptomatically, there would be a low level of perceived susceptibility even among the high-risk population. Being unaware that CKD cannot be cured with medications could also affect the level of perceived seriousness in the individual.

Patient characteristics such as age, ethnicity of a minority group, education level and socioeconomic status were also found to be associated with late referrals to a nephrologist for CKD. Late referrals have been found to be associated with poorer outcomes [19]. Therefore, low knowledge levels of chronic kidney disease would not only affect the success of primary and secondary disease prevention, it could also affect the management of CKD.

There were some limitations to our study. Firstly, our survey was a cross sectional study and conducted using a convenience sample of general public respondents who sought attendance at 3 primary health care centres and therefore, not a true representative sample of the general population per se. While our sampled population had similar percentage distribution to the general population in terms of gender and ethnicity, our study results are considered preliminary and cannot be extrapolated to foreigners due to the small number of foreigners in our sampled population. There is also a possibility of selection bias of respondents who might be more involved in self-management who have volunteered to participate in this survey, thus resulting in a higher level of knowledge of CKD as compared to the general population. We have tried to mitigate this bias by recruiting consecutive respondents for the survey. Secondly, underlying comorbidities were self-reported and clinical data of respondents with diabetes and hypertension were unavailable. Therefore, we were unable to exclude those with undiagnosed CKD or evaluate the relationship between knowledge of CKD and awareness of CKD.

Further research could be conducted using a sample of the general population with a more refined questionnaire regarding general knowledge of CKD in order to better assess the knowledge levels of the general public. At the same time, research could be done to evaluate interventions that might improve the knowledge of CKD of particularly those who are at risk of developing CKD.

## Conclusions

With an increasing incidence of chronic kidney disease, our study suggests that knowledge of chronic kidney disease is reduced among the vulnerable population like those in the lower socioeconomic groups. Education efforts could be more targeted towards particularly those at risk of developing chronic kidney disease at the primary care level with more emphasis paid to older respondents with lower education levels and of lower income.

## Appendix

### Annex 1. Screening questions

1. **Are you above 21 years old?**

□ Yes □ No **If Yes, continue completing the survey**

2. **Are you currently on dialysis treatment or did you receive a kidney transplant?**

□ Yes □ No **If Yes, do not complete the survey**

3. **Can you please choose the language that you prefer to use during this survey?**

□ English □ Chinese □ Malay

### Annex 2. Questions on chronic kidney disease

Please tick the best option for each of the following questions.

1. **How many healthy kidney(s) does a person need to lead a normal life?**

□ 1. One

□ 2. Two

□ 3. I don’t know.

2. **What is the function of a kidney in a human body?**

□ 1. To break down food.

□ 2. To produce substances that break down fats.

□ 3. To filter waste products in the blood.

□ 4. I don’t know.

1. 3. **What can cause kidney disease?**

□ 1. High blood pressure

□ 2. Diabetes

□ 3. Inherited condition

□ 4. All of the above

□ 5. I don’t know

4. **What are the symptoms of early kidney disease that might progress to kidney failure?**

□ 1. Bubbles in the urine

□ 2. Back pain

□ 3. Blood in the urine

□ 4. Can present without any symptoms/ complaints

□ 5. All of the above

□ 6. I don’t know

5. **Which of the following statement about kidney disease is INCORRECT:**

□ 1. Kidney disease can be prevented.

□ 2. Kidney disease can be cured with medications.

□ 3. A person is said to have kidney disease when he/she needs dialysis.

□ 4. None of the above

□ 5. I don’t know

6. **Where can dialysis treatment be carried out?**

□ 1. In a dialysis centre or at home.

□ 2. Only in a dialysis centre.

□ 3. Only at home.

□ 4. I don’t know.

7. **What is the best medical treatment for End Stage Kidney Failure?**

□ 1. Medication.

□ 2. Dialysis.

□ 3. Kidney transplant.

□ 4. I don’t know.

## Competing interests

The authors declare that they have no competing interests.

## Authors’ contributions

CWL was involved in conception and design, acquisition of data, analysis and interpretation of data as well as the writing of the manuscript. VD and TAS contributed to the analysis and interpretation of data and writing of the manuscript. SVDE was involved in conception and design, acquisition of data as well as analysis and interpretation of data. JLFY was involved in conception and design, interpretation of data as well as the drafting of the manuscript. STS, STHH, GTCS, PGSL and CL were involved in the design of the study as well as the interpretation of data. TKYS was involved in conception and design of the study, acquisition of data, interpretation of data as well as the writing of the manuscript. All authors have read and given their approval of the manuscript.

## Pre-publication history

The pre-publication history for this paper can be accessed here:

http://www.biomedcentral.com/1471-2369/13/54/prepub
